# The emerging role of insulin-like growth factor 1 receptor (IGF1r) in gastrointestinal stromal tumors (GISTs)

**DOI:** 10.1186/1479-5876-8-117

**Published:** 2010-11-15

**Authors:** Maria A Pantaleo, Annalisa Astolfi, Margherita Nannini, Guido Biasco

**Affiliations:** 1 Department of Hematology and Oncological Sciences "L.A.Seragnoli", S.Orsola-Malpighi Hospital, University of Bologna, Italy; 2 Interdepartmental Centre of Cancer Research "G. Prodi", University of Bologna, Italy

## Abstract

Recent years have seen a growing interest in insulin-like growth factor 1 receptor (IGF1R) in medical oncology. Interesting data have been reported also on IGF1r in gastrointestinal stromal tumors (GISTs) especially in children and in young adult patients whose disease does not harbour mutations on KIT and PDGFRA and are poorly responsive to conventional therapies. However, it is too early to reach conclusions on IGF1R as a novel therapeutic target in GIST because the receptor's biological role is still to be defined and the clinical significance in patients needs to be studied in larger studies. We update and comment the current literature on IGF1R in GISTs and discuss the future perspectives in this promising field.

## Introduction

**Figure 1:**
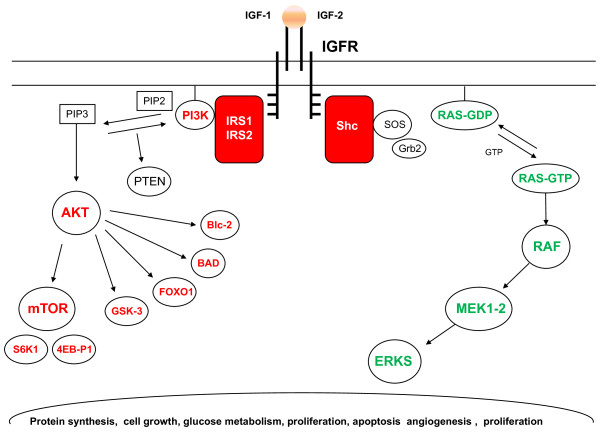
**IGF1R pathway**.

Recent years have seen a growing interest in insulin-like growth factor 1 receptor (IGF1R) in medical oncology. IGF1R is a tyrosine kinase receptor that binds both IGF1 and IGF2 [1]. After ligand binding, the tyrosine kinase domain is activated and stimulates the intracellular signaling pathways that control the proliferation rate and apoptosis (Figure 1). Two key signal-transduction networks have been identified: GPTase Ras-Raf-ERK/MAPK and PI3K-AKT/mTOR [2]. The IGF system plays a key role in the growth and development of normal tissue. However, aberrations of this molecular pathway such as overexpression of IGF1R, elevated plasma levels of IGF1, loss of IGF2 imprinting, or genetic polymorphisms of the gene encoding IGF1 have been found in many cancers, affecting multiple aspects of malignancy such as tumor growth and metastases [3,4]. The biologic role of the IGF system in rhabdomyosarcomas, neuroblastomas, osteosarcomas and soft-tissue sarcomas has been widely demonstrated by preclinical and clinical evidence [5-20]. The IGF1R pathway has also been shown to exhibit cross-talk with a number of other signaling pathways such as EGFR and HER2, suggesting a possible role in mediating resistance to drugs targeting these molecules [21,22]. Therefore IGF1R has been investigated in cancer therapy and strategies for its inhibition in sarcoma have already been reported [23-26]. Inhibition of IGF1R affects Ewing's sarcoma cell growth *in vivo *[27,28] and seems to sensitize sarcoma cells to conventional agents by a synergistic interaction, suggesting a therapeutic combination approach [29,30]. Although the family of sarcomas is the most investigated field, aberrant IGFIR signaling has been implicated in other solid tumors, including lung, breast and colon cancer [31-35]. Interesting data have been reported on IGF1R in gastrointestinal stromal tumors (GISTs) [36-40]. Current literature on IGF1R in GISTs needs to be updated with a discussion on future perspectives in this field.

As is well known, GISTs are characterized by the abnormalities of the KIT and PDGFRA receptors that represent the key oncogenic event and most important therapeutic target [41-45]. In a small subset of patients the disease does not present any mutation and is defined as wild-type (WT). The mutational status of KIT and PDGFRA affects response to tyrosine kinase inhibitors and confers primary or secondary resistance [44,45]. Recently, IGF1R has emerged as a novel molecular signaling pathway other than KIT and PDGFRA on GISTs [36-40]. Tarn and colleagues evaluated IGF1R with SNPs array, FISH and realtime PCR at genomic level, and with western blotting (WB) and immunohistochemistry (IHC) at protein level [36]. By SNPs analysis they found that the IGF1R gene was amplified especially in WT GISTs compared with mutant GISTs, including a pediatric case. To determine whether enhanced expression of IGF1R is associated with gene amplification, they evaluated IGF1R gene copy number in mutant and WT GISTs using a genomic-based quantitative PCR assay. Seven of the 10 WT GISTs had the IGF1R amplification (copy number range, 2.5-4 copies) compared with only 5 out of 18 mutant GISTs (P = 0.04). IGF1R gene amplification was also confirmed by FISH. No mutations in IGF1R gene were found in the WT GISTs. The protein level was abundantly expressed only in WT GIST by WB and IHC (Cell Signaling antibody). Agaram and colleagues evaluated IGF1R in 17 patients as gene expression profiling (mRNA level) and found that it was up-regulated in children and young adults (patients < 30 years old) [37]. We examined the IGF1R status in 8 patients with gastric GIST [38]. IGF1R was studied as gene expression profiling performed with Affymetrix GeneChip HG-U133 Plus 2.0 arrays and as genomic copy number with SNP array analysis Affymetrix Genome Wide Human SNP 6.0 arrays, and at protein level with IHC (Santa Cruz Biotechnology Inc). The unsupervised analysis of gene expression profiling in our patients merged with a data set from a gastric GIST showed that IGF1R was up-regulated in two young patients ( < 30 years old) with both WT disease and metastases at diagnosis, and was confirmed by WB and IHC. SNPs array analysis of the genomic copy number showed that neither of the 2 young patients had tumors with IGF1R amplification. More recently, Janeway and colleagues studied IGF1R with WB, SNP and FISH and found a strong expression of the receptor in 8 out of 9 WT pediatric GISTs [39]. By SNP analysis, none of the pediatric WT GISTs had IGF1R amplification. To validate the SNP data, FISH was done in two patients and in one additional pediatric WT GIST for which there was insufficient fresh frozen specimen for SNP analysis and no gene amplification was documented in any of the 3 cases. Lastly, Braconi and colleagues evaluated IHC expression of IGF1R (Santa Cruz antibody) and its ligands IGF1 and IGF2 in 94 patients [40]. They found that the IGF1R was strongly expressed in most cases both WT and mutant, but the ligands showed different levels of expression.

## Discussion

Despite the above studies, it is too early to reach conclusions on IGF1R as a novel therapeutic target in GIST. Firstly, the data from these studies are related to different levels of biological information, and secondly they were obtained using different assays, different antibodies and different scores. In addition, although we cannot generalize, longstanding experience of EGFR in colorectal cancer as a target and molecular predictor of EGFR inhibitors should be considered before talking about novel targets in medical oncology [46,47]. Moreover, to date few data have been reported on IGF1R in GISTs and the receptor's true role in the pathogenesis of the disease remains to be defined. As a consequence, the clinical implications such as the correlation with mutational receptors status, clinical outcome, prognosis, therapeutic responsiveness or the exact GIST population with IGF1R deregulation require further investigation.

First of all, the mechanism by which IGF1R is strongly expressed in WT GISTs has not been identified. Low level amplification in 6 WT GISTs was reported only by Tarn and colleagues [36], whereas the other reports on IGF1R [38,39] and SNP-array data [48,49] that collectively analyzed 26 pediatric or young adult WT GIST cases showed no gain at chromosome 15. Hence it is conceivable that IGF1R amplification represents a rare event in WT GISTs, and that IGF1R overexpression is reasonably sustained by other mechanisms. The lack of genomic amplification is not surprising, since IGF1R is not generally found amplified in human tumors [1,24]. Many mechanisms contribute to IGF1R overexpression in sarcomas [24] such as receptor upregulation or overexpression of ligands driven by multiple mechanisms like fusion genes (PAX3-FKHR; EWS-WT1; EWS-FLI1), loss of imprinting (LOI) of IGF2, or loss of tumor suppressor genes (WT1, PTEN, p53). IGF2 LOI deserves further investigation in WT GISTs because it is an important mechanism in many pediatric solid tumors, and because ligand expression is found in WT GISTs [40].

The most exciting future perspectives are first to study the biological role of IGF1R in GISTs in *in vitro *and *in vivo *models, and second to investigate the receptor's clinical significance further using ex-vivo analyses (IHC, gene expression, SNP, etc) in larger series of patients. About the biological role, notwithstanding the very high expression of IGF1R in GIST carrying a wild type KIT and PDGFRA status, suggesting a possible role as a therapeutic target, almost no experimental data are available on the functional role and oncogenic relevance of this receptor in GIST tumors. The only data were reported by Tarn and colleagues who treated GIST-T1 and GIST 882 cell lines with the IGF1R inhibitor NVP-AEW541, measuring an IC_50 _of 3.7 - 3.9 μM [36]. Albeit encouraging, this result is not predictive of any activity in GIST WT tumors, since these cell lines poorly express IGF1R, harbor KIT mutations and are dependent on aberrant KIT signaling for proliferation and survival. In addition, the IC_50 _concentration is suggestive of the inhibition of tyrosine kinase targets other than IGF1R [50]. IGF1R signaling was blocked in many other types of sarcomas to explore its role in cell proliferation and survival *in vitro*, and tumor growth, invasion and metastasis *in vivo *in animal models [25]. Unfortunately preclinical studies assessing the relevance of IGF1R in GISTs are hampered by the lack of a suitable *in vitro *model of WT GIST. To overcome this problem KIT-mutant GIST cell lines could be infected with IGF1R vectors inducing IGF1R expression and analyzing its effect on cell growth, proliferation, apoptosis and response to agonists (IGF1 and IGF2) and IGF1R-inhibitors or antibodies [51]. IGF1R induction could also be coupled with KIT downregulation to explore the relationship between the two oncogenic signaling pathways. IGF1R-transfected GIST cell lines could also be used *in vivo *in suitable xenograft animal models to test the efficacy of different IGF1R-inhibitors and the effect of the combination with standard front line therapies [52]. These analyses are particularly necessary to confirm the putative oncogenic role of IGF1R in WT GISTs. Indeed the possibility that IGF1R is not a tumor-specific target, but just a stage-specific differentiation marker of interstitial cell of Cajal (ICC) precursors cannot be ruled out, since a recent work by Lorincz and colleagues showed that ICC precursors are a rare IGF1R-positive, Kit^(low)^, CD44^(+)^, CD34^(+)^, Insr^(+) ^cell population, retained in postnatal life, that is dependent on IGF signaling for survival and differentiation [53]. The absence of IGF1R activating mutations or genomic amplifications in WT GIST does not offer even indirect support of a dominant oncogenic role [37-39]. Besides functional *in vitro *and *in vivo *studies, in-depth analysis of WT GISTs genomic and transcriptomic profile by microarray or next generation sequencing techniques will help to clarify IGF1R's role as a marker or therapeutic target, and the mechanism of its over-expression in this rare subtype of GIST that is poorly responsive to conventional therapies [37,48,49].

If preclinical functional studies demonstrate the pathogenetic role of IGF1R in WT GISTs, the IGF axis blockade may be beneficial in the treatment of GIST. However, in-depth analysis of the IGF axis in GISTs is mandatory, since ligand signaling could also be driven by other receptors like insulin receptor isoform A (IR-A), that is especially overexpressed in cancer [54], and whose expression and function have not been investigated in GISTs. Commonly, membrane receptor blockade can be achieved with monoclonal antibodies that block the extracellular domain, or with tyrosine kinase inhibitors that block the intracellular tyrosine kinase. In theory, if they work both should block receptor activation, and thereby block the intracellular pathways. Of course, direct inhibition of the molecules of these pathways, such as MAPK or PI3K or mTOR, is a potential therapeutic option especially because no amplification or kinase mutation have been identified for IGF1R. Moreover, this strategy may have an enhanced antitumor effect since MAPK, PI3K or mTOR may also be activated by KIT and PDGFRA receptors and may overcome KIT and PDGFRA-dependent imatinib resistance [55].

Glycemic derangements related to insulin-like growth factors such as the pro-IGF-IIE and insulin-like growth factor-binding proteins have been described in GISTs, and they may become more important in patient management because of a potential cross-reactivity between IGF1R and the insulin receptor [56-59]. Even though metabolic derangements are uncommon and no data are available on what might happen to glucose metabolism after administration of IGF1R-targeted drugs, great attention should be paid to these clinical aspects and caution exerted during therapeutic IGF1R inhibition in GIST.

## Conclusions

In conclusion, a novel signaling pathway other than KIT and PDGFRA is emerging in GISTs, and more preclinical studies are needed to disclose its biological role. Larger population studies are warranted to identify patients who may benefit from IGF1R inhibitors such as children or also young adult WT patients. Moreover, these analyses should be centralized as was done for KIT and PDGFRA mutational status especially because GIST is a rare disease.

## Abbreviations

(IGF1R): Insulin-like growth factor 1 receptor; (GISTs): Gastrointestinal stromal tumors; (PDGFRA): Platelet derived growth factor receptor; (WB): Western blotting; (IHC): Immunohistochemistry; (WT): Wild-type

## Competing interests

The authors declare that they have no competing interests.

## Authors' contributions

MAP and GB: concept and design. MAP, AA and MN: writing. AA and MN: literature analysis. All authors gave final approval.
